# Motor improvement estimation and task adaptation for personalized robot-aided therapy: a feasibility study

**DOI:** 10.1186/s12938-020-00779-y

**Published:** 2020-05-14

**Authors:** Christian Giang, Elvira Pirondini, Nawal Kinany, Camilla Pierella, Alessandro Panarese, Martina Coscia, Jenifer Miehlbradt, Cécile Magnin, Pierre Nicolo, Adrian Guggisberg, Silvestro Micera

**Affiliations:** 1 Bertarelli Foundation Chair in Translational Neuroengineering, Center for Neuroprosthetics and Institute of Bioengineering, School of Engineering, École Polytechnique Fédérale de Lausanne (EPFL), 1015 Lausanne, Switzerland; 2 Institute of Bioengineering/Center for Neuroprosthetics, Ecole Polytechnique Fédérale de Lausanne (EPFL), Lausanne, Switzerland; 3 Department of Radiology and Medical Informatics, University of Geneva, Geneva, Switzerland; 4 Translational Neural Engineering Area, The Biorobotics Institute, Scuola Superiore Sant’Anna, 56025 Pisa, Italy; 5 Wyss Center for Bio- and Neuro-Engineering, 1202 Geneva, Switzerland; 6 Brain Electrophysiology Attention Movement Laboratory, Institute of Psychology, University of Lausanne, Lausanne, Switzerland; 7 Division of Neurorehabilitation, Department of Clinical Neurosciences, University Hospital Geneva, Geneva, Switzerland; 8 Laboratory of Cognitive Neurorehabilitation, Department of Clinical Neurosciences, Medical School, University of Geneva, Geneva, Switzerland

**Keywords:** Personalized therapy, Rehabilitation robotics, Stroke rehabilitation

## Abstract

**Background:**

In the past years, robotic systems have become increasingly popular in upper limb rehabilitation. Nevertheless, clinical studies have so far not been able to confirm superior efficacy of robotic therapy over conventional methods. The personalization of robot-aided therapy according to the patients’ individual motor deficits has been suggested as a pivotal step to improve the clinical outcome of such approaches.

**Methods:**

Here, we present a model-based approach to personalize robot-aided rehabilitation therapy within training sessions. The proposed method combines the information from different motor performance measures recorded from the robot to continuously estimate patients’ motor improvement for a series of point-to-point reaching movements in different directions. Additionally, it comprises a personalization routine to automatically adapt the rehabilitation training. We engineered our approach using an upper-limb exoskeleton. The implementation was tested with 17 healthy subjects, who underwent a motor-adaptation paradigm, and two subacute stroke patients, exhibiting different degrees of motor impairment, who participated in a pilot test undergoing rehabilitative motor training.

**Results:**

The results of the exploratory study with healthy subjects showed that the participants divided into fast and slow adapters. The model was able to correctly estimate distinct motor improvement progressions between the two groups of participants while proposing individual training protocols. For the two pilot patients, an analysis of the selected motor performance measures showed that both patients were able to retain the improvements gained during training when reaching movements were reintroduced at a later stage. These results suggest that the automated training adaptation was appropriately timed and specifically tailored to the abilities of each individual.

**Conclusions:**

The results of our exploratory study demonstrated the feasibility of the proposed model-based approach for the personalization of robot-aided rehabilitation therapy. The pilot test with two subacute stroke patients further supported our approach, while providing encouraging results for the applicability in clinical settings.

*Trial registration* This study is registered in ClinicalTrials.gov (NCT02770300, registered 30 March 2016, https://clinicaltrials.gov/ct2/show/NCT02770300)

## Background

With the increase of life expectancy, it is estimated that stroke-related impairments will be ranked fourth most important cause of disability in Western countries by 2030 [1]. Despite early rehabilitative interventions, 55% to 75% of the patients still suffer from upper limb impairments in the chronic state of the injury [2–4]. The recovery of reaching and grasping movements is therefore a crucial therapeutic goal in stroke rehabilitation [5].

Post-stroke rehabilitation usually relies on task-oriented repetitive movements that help improving motor function and training new control strategies. In this regard, the amount of goal-directed and challenging practice, rather than daily intensity alone, seems to be the most effective factor in neurorehabilitation [6]. In the last two decades, robot-aided motor training has shown potential for the recovery of lost motor abilities in upper limbs after stroke [7–9]. While providing intense and highly repeatable motor training, robotic devices also offer means to control and quantify movement performance. Despite this strong potential, controlled clinical trials have so far not been able to confirm whether robotic therapy is more effective than conventional methods in restoring motor abilities [10–12]. It has been argued that this might be related to saturation effects in the patients’ motor performances and a lack of automatic methods to promptly detect them [13]. Indeed, a recent review analyzing 38 studies on this topic [14] concluded that robotic therapy had rather small effects on patients’ motor control compared to other interventions.

The automatic and personalized adaptation of the rehabilitation training has been suggested as a pivotal step to improve the outcome of robot-aided rehabilitation and the clinical relevance of such solutions [15]. As a matter of fact, motor learning is known to be maximized when the difficulty level of the training task matches the patient’s level of ability [16]. Recent advances in the field of personalized robotic rehabilitation have therefore focused on the design of customized training protocols, including individualized selection of upper limb movements [17]. One of the pivotal aspects underlying the development of a personalized rehabilitation training is the definition of performance measures that can correctly capture the different aspects of motor recovery, as well as their specific dynamics. Different measures have been used to assess the patient’s “status” during training (i.e., motor performance, engagement, etc.) in order to adjust the proposed tasks accordingly. Kinematic performance measures, such as movement accuracy, smoothness, velocity, inter-joint coordination, range of motion and stiffness [18–24], game-related statistics [13, 25], measures of muscle activity [18], or the combination of kinematic and psychophysiological measurements [26–28] have been among the measures used for the design of patient-tailored training protocols. However, those approaches either focused on a single performance measure describing a specific aspect of rehabilitation or used multiple measures, but lacked the ability to meaningfully synthesize the information from these variables. Integrating this information into a single measure, yet representative of the patient’s multidimensional rehabilitation response, would facilitate the monitoring of the multifaceted progress of the patient and provide a way to trigger task adaptation while enormously simplifying the design of personalized rehabilitation training.

A first approach addressing this issue was presented in our previous work [29]. Previously, we have used a state-space model to merge the information from different kinematic measures and, in this way, estimated motor improvement (MI) of chronic stroke patients exercising with a planar robotic device for upper limb rehabilitation. In this previous work [29], we used four performance measures to estimate the MI: (1) the movement velocity (MV); (2) the movement accuracy (nMD); (3) the movement smoothness (nPK); (4) the percentage of successful tasks executed during each session (%SUCC). Following post hoc analyses on the recorded performance measures, we showed that such model would be capable of mimicking decision rules applied by physical therapists regarding the adaptation of the task difficulty. In most cases, the model even appeared to be faster than the therapists in detecting when the patients’ motor performance had reached a plateau and when more challenging tasks should have been proposed. Yet an automatic task adaptation based on such a model was lacking from our previous implementation.

In the current study, we therefore build on these results to implement a method able to continuously detect patient’s motor improvement and adapt the training task for three-dimensional movements using an upper-limb exoskeleton. Indeed, most of the adaptive approaches mentioned before were restricted to planar workspaces, hindering their applications to functional movements exploring three-dimensional workspaces that better resemble those performed during daily life activities. Evaluating and estimating motor improvement is particularly complex in three-dimensional training workspaces, where the visual evaluation of motor performance becomes more challenging. Under these circumstances, a method able to autonomously estimate patient training progress, in particular for movements in different directions, could provide fundamental support to therapists. In contrast to our previous work, here we employed a continuous implementation of the motor improvement estimation and the training adaptation routine. Indeed, the immediate task adaptation within the same training sessions could not only increase patients’ engagement, but also foster their attention control, possibly leading to improved reaching performances [30].

However, in order to enable the use of such methods for clinical applications, it is first necessary to validate their feasibility and safety under controlled experimental conditions. The main objective of this exploratory study was hence to demonstrate feasibility and safety for the proposed method and to comprehend whether such approach could possibly be applied in clinical settings. We, therefore, devised an experiment to test our model in a group of healthy subjects. In order to mimic the recovery of motor performance observed in stroke patients, we inverted the visual feedback for the point-to-point reaching movements that the healthy subjects had to perform in a three-dimensional training environment using a robotic upper-limb exoskeleton. Previous studies on visually manipulated motor tasks showed that most people could cope with similar manipulations after training [31–35]. Accordingly, we hypothesized that performances would drop after the introduction of the inverted visual feedback (i.e., movements would become slower and jerkier), but would then gradually improve and eventually reach a plateau—with temporal dynamics resembling the ones occurring in robot-aided rehabilitation of stroke patients [29, 36, 37]. Moreover, previous work has demonstrated that for reaching movements in planar setups, participants showed better performance for targets lying on the axis perpendicular to the inversion [32, 38]. Though in this study we used a three-dimensional setup, we also hypothesized that participants would have less difficulties with the targets lying on one of three coordinate axes (on-axis targets), as they involved inversions in only one dimension (in contrast to inversions in two dimensions for off-axis targets, i.e., targets not lying on the axes). Using this setup, we tested whether our model was capable of tracking individual motor improvement induced by motor adaptation, and whether it was able to personalize the training by identifying “recovered” (i.e., adapted) movements in real-time.

To provide further evidence about the clinical usability of the presented approach, we finally performed a pilot test with two subacute stroke patients. The objective of this pilot test was to evaluate the model in an authentic clinical context and with two patients exhibiting different degrees of motor impairment. The patients underwent 4 weeks of robot-aided rehabilitation training, performing the same point-to-point reaching movements as the healthy subjects. In this case however, visual feedback was provided normally, without inversion. We hypothesized that the model would suggest two distinct training adaptation schemes for the patients, optimizing their motor recovery by proposing reaching movements that match the individual abilities of each patient.

## Results

### Experimental validation with healthy participants

**Fig. 1:**
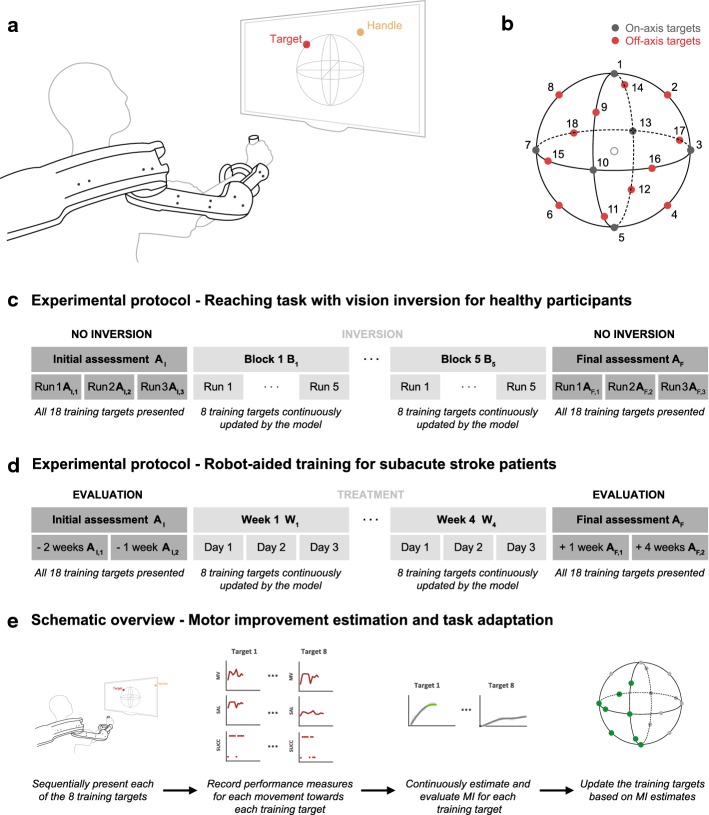
Experimental setup and protocols. **a** Schematic overview of experimental setup. **b** Design of the three-dimensional point-to-point reaching task. Eighteen targets (representing the different subtasks) are positioned over a sphere of 19 cm of radius (equally distributed on the three planes). The empty circle represents the center of the workspace (starting position). **c** Experimental protocol for healthy participants. Experiments were completed in a single session and were divided into blocks (one initial assessment block A_I,1–3_, five inversion blocks B_1–5_, one final assessment block A_F,1–3_). The assessment blocks consisted of three runs, each composed of 18 reaching movements (one towards each target). The inversion blocks consisted of five runs, each composed of eight reaching movements. The training targets for the inversion blocks were automatically selected by the implemented personalization routine. Breaks were allowed between the blocks to prevent fatigue. **d** Experimental protocol for the patient. During the initial (A_I,1–2_) and final (A_F,1–2_) assessment sessions, all 18 targets were presented to the patient. For each treatment session eight training targets were selected by the implemented personalization routine. The total number of repetitions performed in each session was determined by the physical therapist. **e** Schematic overview of the different steps performed for the adaptive scheduling of the reaching task with vision inversion for healthy participants and the reaching task without vision inversion for patients

The model was first tested with 17 healthy subjects, who performed a point-to-point reaching task using an upper-limb exoskeleton (ALEx, Fig. 1a, b). As an additional constraint, the healthy subjects had to complete the task with a vision inversion implemented in the blocks B_1–5_ (Fig. 1c). During these five blocks, we tested whether our model was capable to continuously adapt the reaching task according to the performances of each individual. For each participant, the performance measures recorded by the exoskeleton were deployed in a state-space model to continuously estimate the motor improvement (MI) for each direction of movement independently. When the MI values for a movement direction passed a certain threshold and remained stable for a given time window (see “Training adaptation routine” section), the movement was replaced with a new target from the training queue (Fig. 1e). The latter contained 18 directions of movement (i.e., targets) and it was generated for each individual at the beginning of the training based on a semi-randomized procedure (see “Healthy participants” section).

#### Task adaptation at subject level

**Fig. 2:**
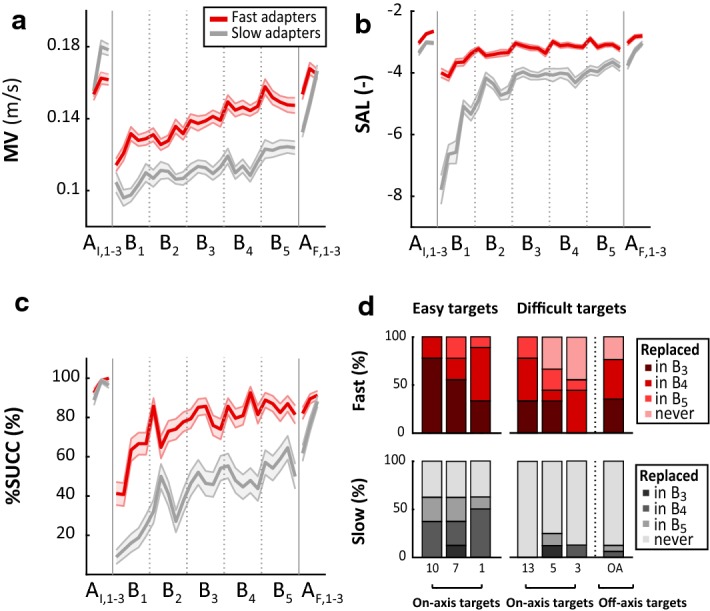
Analysis of performance measures for the experiment with healthy participants. Average values of mean velocity (MV, panel **a**), spectral arc length (SAL, panel **b**) and rate of success (%SUCC, panel **c**) for each run (eight reaching movements) of fast (red) and slow (grey) adapters. Measures were averaged for all targets presented during a run and for all subjects of a group. Shaded areas depict standard error of the mean (sem). Vertical bars (panel **d**) depict the percentage of subjects in each group for which a target was replaced in B_3–5_ or was not replaced at all. No targets were replaced in and B_1–2_ due to lack of data needed for proper estimation of motor improvement

Despite a general improvement for all participants, the healthy subjects differed considerably in their adaptation speed, as quantified by the number of new targets introduced during the inversion blocks B_1–5_. Since this study was exploratory in nature, we did not expect a priori such a variety of adaption speeds. However, a post hoc analysis of the number of new introduced targets allowed to identify two groups of participants using a median split. Specifically, participants were classified into fast adapters (*n* = 9, 7.7 ± 1.1 new targets) and slow adapters (*n* = 8, 2.6 ± 2.0 new targets). This result emerged as an unforeseen opportunity to highlight the model’s capability to differentiate varying motor adaptation rates. As hypothesized, the performance measures [i.e., movement velocity (MV), movement smoothness (SAL) and task completion rate (%SUCC)] degraded for both groups after the introduction of the vision inversion in B_1_ (Fig. 2a–c).

Participants in both groups gradually improved from B_1_ to B_5_, although they did not reach their initial motor performances (i.e., performances during A_I,1–3_). Friedman tests confirmed the differences between the blocks A_I,1–3_, B_1_ and B_5_, for both groups and for all performance measures (Table 1). Post hoc analyses were performed using Wilcoxon signed-rank tests with Holm–Bonferroni corrections for three comparisons. The analyses confirmed pairwise differences between different pairs of blocks (A_I,1–3_ and B_1_, B_1_ and B_5_, A_I,1–3_ and B_5_,) within both groups and for all performance measures, except for MV between A_I,1–3_ and B_5_. When introduced to the vision inversion in B_1_, fast adapters outperformed slow adapters as measured by all performance measures. A statistical comparison of the performance measures in A_I,1–3_ between the two groups (Table 2) showed that there were no statistically significant differences for SAL and %SUCC (statistical power of 0.99 and 0.85, respectively), while no conclusions could be drawn for MV (statistical power 0.42). The performance difference was still observable in B_5_, the last block with vision inversion.

**Table 1:** Within-group comparisons of healthy subjects at three different time points

	Performance measures (mean ± standard error of the mean, sem)			Friedman’s test		Wilcoxon signed-rank test with Holm–Bonferroni correction (for three comparisons)		
	A_I,1–3_	B_1_ (inversion)	B_5_ (inversion)	Chi-square (2)	*P* value	*p*_corr_ A_I,1–3_–B_1_	*p*_corr_ B_1_–B_5_	*p*_corr_ A_I,1–3_–B_5_
Fast adapters (*n* = 9)								
MV (m/s)	0.16 ± 0.01	0.13 ± 0.01	0.15 ± 0.01	10.89	0.0043	0.0156	0.0117	0.3594
SAL	− 2.81 ± 0.07	− 3.77 ± 0.12	− 3.09 ± 0.19	16.22	0.0003	0.0117	0.0078	0.0117
%SUCC	97.1 ± 1.1	55.8 ± 3.4	85.4 ± 2.8	18.0	0.0001	0.0117	0.0117	0.0117
Slow adapters (*n* = 8)								
MV (m/s)	0.17 ± 0.01	0.10 ± 0.01	0.12 ± 0.01	13.0	0.0015	0.0156	0.0156	0.1094
SAL	− 3.13 ± 0.10	− 6.39 ± 0.34	− 3.82 ± 0.11	16.0	0.0003	0.0234	0.0234	0.0234
%SUCC	94.7 ± 1.5	16.3 ± 3.9	57.0 ± 5.6	16.0	0.0003	0.0234	0.0234	0.0234

**Table 2:** Between-group comparisons of healthy subjects at three different time points

	Fast adapters (*n* = 9)	Slow adapters (*n* = 8)	Wilcoxon rank-sum test with Holm–Bonferroni correction (for three comparisons)
Performance measures in A_I,1–3_ (mean ± sem)			*p*_corr_
MV (m/s)	0.16 ± 0.002	0.18 ± 0.003	0.2359
SAL	− 2.69 ± 0.03	− 3.03 ± 0.04	0.0619
%SUCC	99.3 ± 0.5	97.3 ± 1.0	0.2973
Performance measures in B_1_ (mean ± sem)			*p*_corr_
MV (m/s)	0.11 ± 0.002	0.10 ± 0.002	0.0360
SAL	− 3.77 ± 0.06	− 6.34 ± 0.18	0.0002
%SUCC	55.6 ± 2.9	16.0 ± 2.3	0.0002
Performance measures in B_5_ (mean ± sem)			*p*_corr_
MV (m/s)	0.15 ± 0.002	0.12 ± 0.002	0.0360
SAL	− 3.09 ± 0.04	− 3.82 ± 0.07	0.0002
%SUCC	85.4 ± 2.1	57.3 ± 3.1	0.0002

#### Task adaptation at subtask level

We then analyzed which initial training targets were replaced by the algorithm during the inversion blocks and when this replacement occurred (Fig. 2d). The insertion of new targets did not start before B_3_, as in B_1–2_ the amount of data for each training target was not sufficient to obtain proper MI estimations (see “Motor improvement model” section). Overall, movements towards the off-axis targets (Fig. 1b) seemed to be more difficult: the algorithm replaced these targets for 13% of the slow adapters and for 77% of the fast adapters. The on-axis targets instead, were replaced for 38% of the slow adapters and 87% of the fast adapters. However, we also observed differences within the on-axis targets: targets 3, 5 and 13 were replaced for 13% of the slow adapters and for 74% of the fast adapters, while the replacement for targets 1, 7 and 10 was achieved by 63% of the slow adapters and by 100% of the fast adapters. Following this analysis, we classified the targets into easy (1, 7 and 10) and difficult (3, 5, 13 and off-axis) subsets. The results suggested that despite the differences in the overall performance, the subsets of easy and difficult targets appeared to be similar for both groups. Nevertheless, we observed an earlier replacement of the easy targets for the fast adapters: 56% of the easy targets were replaced in B_3_ (4% for slow adapters), 33% were replaced in B_4_ (38% for slow adapters), and 11% were replaced in B5 (21% for slow adapters). In contrast, for the difficult targets, the fast adapters also needed more time to achieve a replacement (if they were replaced eventually): 26% of the difficult targets were replaced in B_3_ (3% for slow adapters), 35% were replaced in B_4_ (5% for slow adapters) and 14% were replaced in B_5_ (5% for slow adapters).

**Fig. 3:**
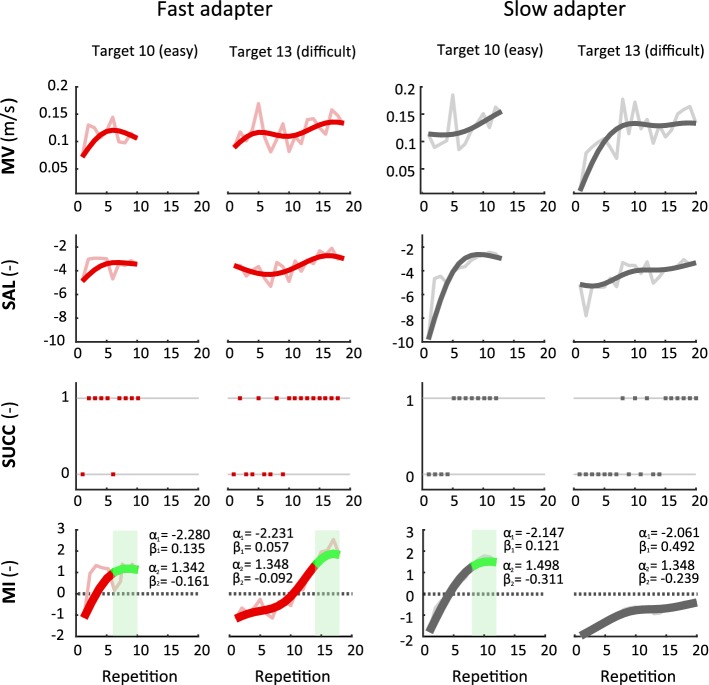
Examples of MI estimates and performance measures at subtask level. Data are presented for a fast adapter and a slow adapter for the same two targets. Repetitions for each target are concatenated for all inversion blocks and presented in chronological order. Data for mean velocity (MV), spectral arc length (SAL) and MI were low pass filtered for visualization purposes (raw data shown in light red/grey). Dotted lines depict one of the necessary conditions (MI > 0) for triggering a target replacement. Green areas indicate the time span where the model detected a performance plateau and triggered a target replacement. Estimated model parameters (*α*_*j*_, *β*_*j*_) for each target and subject are presented next to the corresponding MI curves (a summary and analysis on the model parameters can be found in Additional file 1)

To illustrate the behavior of individual participants at subtask level, we present the data of one exemplary subject from each group for the movements towards the same two targets (Fig. 3). We selected one target from the subset of the easy (target 10) and one target from the subset of the difficult (target 13) targets. The examples illustrate the different adaptation rates observed between subjects and targets. For the easy target, the performance measures for the fast adapter quickly improved and approached a plateau. The slow adapter, instead, showed difficulties until the fourth repetition, reflected particularly by SAL and SUCC. Nonetheless, starting from the fifth repetition, they also managed to adapt the movements to the visual inversion and finally reached the conditions for the target replacement at the twelfth repetition. The difficult target instead, appeared to be more challenging for both subjects. For this target, the fast adapter showed an improvement in all performance measures only after the tenth repetition and finally reached the conditions for the target replacement after 18 repetitions. In contrast, the slow adapter did not manage to satisfy the conditions for a replacement. Despite a trend of improvement, the motor performance was not yet sufficient to trigger a replacement of the target.

### Pilot test with subacute stroke patients

To provide further evidence about the feasibility of the presented approach in clinical settings, we finally performed a pilot test with two subacute stroke patients, who completed 4 weeks of personalized robot-aided training complementing standard rehabilitation therapy (Fig. 1d, see “Subacute stroke patients” section for details). During the robot-aided training, the patients performed the reaching tasks without vision inversion and the set of targets was automatically adapted based on a continuous evaluation of the MI estimates for each training target.

**Fig. 4:**
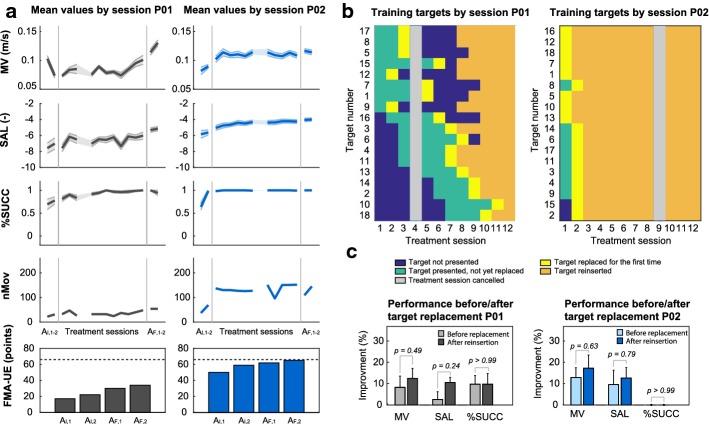
Summary of the results from the pilot test with two subacute stroke patients. **a** The first three rows show the mean values for mean velocity (MV), spectral arc length (SAL) and rate of success (%SUCC) for each assessment and treatment session of both patients. Measures were averaged for all targets presented during a session, shaded areas depict standard error of the mean (sem). The fourth row shows number of movements performed by the patients in each session. The fifth row shows the scores on the Fugl-Meyer scale for upper extremities (FMA-UE) for initial (A_I,1–2_) and final (A_F,1–2_) assessment sessions. The dotted line indicates the maximum achievable score for FMA-UE (66 points). **b** Summary of the training targets presented to the patients in each treatment session. Targets are listed by the order as presented to the patients (first eight targets from the top are the initial training set). **c** Analysis of performance measures for two different time points (before replacement and after reinsertion). Values are compared between the last four movements towards a training target before its replacement and the first four movements towards the target after it has been reinserted for training. The data show the mean improvement for MV, SAL and %SUCC averaged for all targets at both time points. Improvements were calculated with respect to the mean values obtained from the first four movements towards each target in A_I,2_. Error bars depict standard error of the mean (sem). *P*-values of Wilcoxon signed-rank tests are reported above the bars

Based on the initial assessment of their scores on the Fugl-Meyer assessment for upper extremities (FMA-UE), we observed a remarkable difference in the degree of motor impairment of patient P01 (22 points at A_I,2_, Fig. 4a) compared to patient P02 (59 points at A_I,2_). This difference was equally reflected by the number of movements (nMov) performed in the initial assessment sessions, which was notably lower for P01 (31 movements compared to 69 movements for P02 at A_I,2_). The different degrees of initial impairment allowed us to evaluate the feasibility of our approach for two patients exhibiting disparate initial motor abilities.

Following the training, both patients showed improvements for MV, SAL, and %SUCC (Fig. 4a). Comparing the performances between the second initial assessment session A_I,2_ and the first final assessment session A_F,1_ we found that both patients improved on all measures (Table 3). One month after the training, both patients performed a follow-up assessment A_F,2_. During this session, we observed that both patients managed to retain the improvements observed in A_F,1_. These differences were confirmed by Friedman tests between the three sessions (A_I,2_, A_F,1_, and A_F,2_) for both patients, except for the %SUCC measure for P02. This can be related to the fact that the values for %SUCC for patient P02 already started at a very high level (98.6% at A_I,2_) and thus left smaller room for improvement. Post hoc Wilcoxon tests with Bonferroni correction for multiple comparisons (i.e., two pairwise comparisons), confirmed the differences between A_I,2_ and A_F,1_ for the performance measures for both patients. For comparisons between A_F,1_ and A_F,2_ (i.e., differences between end of treatment and follow-up 1 month later), the only statistically significant difference was found for MV values of P01. Though no clear differences could be observed between A_F,1_ and A_F,2_ for the other measures, no further conclusions can be drawn due to the low statistical power of the tests (smaller than 0.8).

**Table 3:** Performance measures of the two stroke patients (P01 and P02) before and after the treatment sessions

	Performance measures by session (mean ± sem)			Friedman’s test		Wilcoxon signed-rank test with Holm–Bonferroni correction (for two comparisons)		
	A_I,2_	A_F,1_	A_F,2_	Chi-square (2)	*P*-value	*p*_corr_ A_I,2_–A_F,1_	*p*_corr_ A_I,2_–A_F,2_	*p*_corr_ A_F,1_–A_F,2_
P01								
MV (m/s)	0.08 ± 0.004	0.11 ± 0.005	0.13 ± 0.005	32.44	9.01e−08	5.89e−04	5.89e−04	0.015
SAL	− 6.92 ± 0.65	− 5.31 ± 0.26	− 5.15 ± 0.23	6.33	0.041	0.035	0.031	0.892
%SUCC	77.8 ± 7.3	100 ± 0.0	94.4 ± 5.6	10.18	0.006	0.031	0.033	> 0.99
P02								
MV (m/s)	0.09 ± 0.003	0.12 ± 0.002	0.11 ± 0.004	18.78	8.36e−05	0.001	0.002	0.586
SAL	− 5.49 ± 0.29	− 4.04 ± 0.21	− 3.99 ± 0.19	16.33	0.0003	0.004	0.003	0.844
%SUCC	98.6 ± 1.4	100 ± 0.0	100 ± 0.0	4.0	0.1353	> 0.99	> 0.99	> 0.99

Along with the improvements of the performance measures, we also observed higher FMA-UE scores for both patients following the training. In that respect, we observed a lower increase for patient P02 (+ 3 points between A_I,2_ and A_F,1_) compared to patient P01 (+ 8 points). Both patients further improved their FMA-UE scores when assessed in the follow-up session A_F,2_. Finally, we also observed an increase in the number of performed movements per session (nMov) for both patients. As for this measurement, patient P02 (+ 40 movements at A_F,1_ and + 76 movements at A_F,2_ compared to A_I,2_) improved more than patient P01 (+ 23 movements at both A_F,1_ and A_F,2_ compared to A_I,2_).

Both patients progressed during the rehabilitation training and eventually achieved a replacement of all 18 training targets. However, the temporal dynamics of these replacements appeared to be strongly different (Fig. 4b). In line with the lower degree of motor impairments observed from the performance measures and the FMA-UE scores, patient P02 achieved a replacement of all training targets after only two training sessions. Patient P01, instead, needed considerably more time to achieve the replacement of all 18 targets. While some of the initial training targets (i.e., targets 9 and 12) were already replaced after two treatment sessions, other targets (i.e., targets 1, 7 and 15) needed more than 4 training sessions to trigger a replacement. It was only after eleven treatment sessions that all eighteen training targets were presented to patient P01. These observations emphasized the ability of our model to differentiate between both subject- and subtask-specific time courses of motor improvement, also in a real clinical setting. The examples illustrate how the model adapted the training schedules according to the patients’ individual abilities, granting patient P01 enough time to practice certain movements, and at the same time, responding to the fast recovery of patient P02 by continuously introducing new training targets.

Upon completion of the full set of training targets (i.e., when all targets had been replaced at least once), the therapy was carried on by reintroducing all targets and presenting them alternatingly in the order in which they were replaced. This allowed us to assess whether the patients’ performance was retained once a training target was reintroduced, so as to validate that the replacements orchestrated by the algorithm had occurred when the movements towards the targets had actually recovered. In order to do so, we compared the mean values for MV, SAL, and %SUCC from the last four repetitions of a movement before a target was replaced by the algorithm with the mean values of the four repetitions of the same movement after the first reinsertion as a training target (Fig. 4c). Both values are calculated with respect to the mean values obtained from the first initial four repetitions of the movements towards a training target. The overall analysis for all 18 targets showed that compared to the initial movements towards the targets, all values for the three performance measures were higher right before the targets were replaced by the algorithm. Moreover, both patients retained the improvements gained during the training or even further improved their performance for a movement when the corresponding training target was reintroduced at a later stage. Using a Wilcoxon signed-rank test, we did not find statistically significant differences (*P* > 0.24) for any value of the three performance measures of both patients between the two time points (i.e., before replacement and after reinsertion). However, no further conclusions can be drawn due to the low statistical power of the tests (smaller than 0.8). Nevertheless, the results indicate that the algorithm only replaced training targets when motor performance had stably improved and that patients’ performances did not degrade when training targets were reintroduced at a later stage.

## Discussion

In this study, we demonstrated the feasibility of a model-based approach for the personalization of robotic rehabilitation training based on motor performance during three-dimensional training tasks. Differently from previous work in this field, the model was designed to allow estimation of motor improvement (MI) in subacute stroke patients, allowing to capitalize on the enhanced potential for plasticity in the early stage after the injury [39, 40]. A first experimental validation in healthy subjects demonstrated the ability of our model to capture MI linked to visual motor adaptation. The results were further validated by a clinical pilot test with two subacute stroke patients, in which motor recovery was tracked and harnessed by our adaptive personalization routine.

### Direction-dependent training adaptation for three-dimensional reaching movements

We first sought to validate the model’s ability to continuously track MI and dynamically adapt the training task under controlled conditions. To this end, we tested our approach in a group of 17 healthy subjects. In order to mimic the motor deficits observed in stroke patients, we introduced a manipulation of the visual feedback, by inverting the directions of the 3D environment. While the physiological mechanisms underlying motor adaptation and motor recovery are most likely not equivalent, the main objective of this experimental design was merely to obtain an adaptation curve that resembles post-stroke motor recovery, on which we could validate the efficacy of our model. Our results suggest that motor adaptation to vision inversions in healthy subjects may exhibit similar temporal dynamics for the selected performance measures as previously observed for stroke patients undergoing robot-aided rehabilitation [29, 41]. Indeed, when introduced to the vision inversion (during the blocks B_1_ to B_5_), the performance of the participants dropped drastically and gradually improved throughout the training (Fig. 2).

During the experiments, the MI model tracked when a movement towards a target was performed efficiently despite the vision inversion, and dynamically adjusted the training by replacing this target with a more difficult one from the training queue. Based on the number of new inserted training targets, we divided the healthy population into two separate clusters: fast and slow adapters. The analysis on the performance measures showed that the fast adapters learned to cope with the manipulated environment very quickly, while the slow adapters needed considerably more time to reach similar performances (Fig. 3). The MI model was able to capture these individual performance differences for different movement directions and introduced new training targets in a well-timed manner, i.e., targets were replaced when subjects reached a performance plateau. The advantages of monitoring motor improvement at subtask level were supported by additional post hoc analyses (see Additional file 1). The analyses illustrated that if motor improvements were estimated for the reaching task overall (i.e., chronologically combining the recorded data for movements in all directions), improvements for individual subtasks would have been obscured by inferior performances of other, more difficult, subtasks. Moreover, the detection of performance plateaus would not correspond to the actual performances for each subtask. As a result, some subtasks would be kept too long, while others would be replaced too soon, potentially leading to a less efficient training schedule. Likewise, individual training progressions for specific subtasks were also observed for the two stroke patients participating in this study (Fig. 4b). We therefore, believe that the current study further supports the approach to specifically consider MI estimation at subtask level, as it has been proposed in our previous work [29]. The results of the current study suggest that this subtask dependency does not only apply to planar movements, but also extends to three-dimensional movements. In order to optimize robotic treatment protocols, future studies should therefore specifically aim at evaluating motor performance at subtask level.

When comparing the performances for each subtask, we observed that off-axis targets were replaced less often than on-axis targets and they, thus, seemed to be more difficult (Fig. 2d). This finding appears to be in line with results from similar studies involving vision inversion in planar setups [32, 38]. These studies demonstrated that participants showed better performance for reaching movements lying on the axis perpendicular to the inversion. The present study extends these insights to three-dimensional reaching movements. However, the results showed that there were also remarkable performance differences among the on-axis targets. An analysis on the replaced training targets demonstrated that the subsets of easy (1, 7 and 10) and difficult (3, 5, 13 and off-axis) targets appeared to be similar for both types of adapters. Easy targets were mostly replaced earlier and more frequently than the difficult ones (Fig. 2d). It could be that the medial and proximal movements towards targets 7 and 10 tended to be easier for the participants. However, since these tendencies were not observed in the patients or the healthy subjects involved in the preliminary study (see Additional file 1), we presume that the performance differences for the on-axis targets could be linked to the visually inverted environment. Previous studies have investigated vision inversion in reaching movements and suggested that the adaptation to such manipulations involves a complex mixture of implicit and cognitive processes [32, 42]. For instance, it has been argued that for reaching tasks involving left–right reversal, new control policies need to be acquired by the motor system, as opposed to visual rotations (i.e., rotating the visual feedback around the movement origin in one direction by less than 90°), which only require a recalibration of an existing control policy [43]. The implicit adaptation to such inversions has previously been assessed by aftereffects [44]. In the current study, we have observed that especially fast adapters had more initial difficulties in readapting their movements when the vision inversion was removed (between B_5_ and A_F,1_). Although this was mainly observed for a few initial movements after removing the inversion, it could suggest that fast adapters were more likely to learn the new control policy through implicit adaptation and therefore, were more successful in completing the inverted reaching task. However, to this day, these phenomena have only been investigated for planar reaching movements, mostly involving a one-dimensional inversion (mirror-reversal). Further research would be necessary to examine these phenomena in three-dimensional reaching movements involving multi-dimensional inversions. In this context, it would also be interesting to determine why the reaching movements towards some on-axis targets appeared to be more challenging in the inverted environment, independent from the individual adaptation speed of the subjects.

Finally, we would also like to raise the question of motivational implications resulting from the automated training adaption. From informal observations made during the experiments with the healthy subjects, we noticed that many participants showed increased motivation and verbalized satisfaction when new training targets were introduced. Motivation is known to be a crucial factor in rehabilitation and finding ways to maintain and improve it has always been a matter of interest [45–47]. With regard to this issue, it seems like the automated character of our approach, enabling dynamic and well-timed task adaptation, may have positive impacts on training engagement.

### Personalization of rehabilitation therapy

The potential of our implementation was finally evaluated in a clinical pilot test with two subacute stroke patients, who completed 4 weeks of robot-aided rehabilitation training following our adaptive approach. Based on the devised method, the training of these two patients was continuously monitored and the point-to-point reaching task was adapted in real-time to match their level of ability.

The results obtained from these two patients suggested that in general, the selected performance measures (MV, SAL and SUCC) appeared to be suitable for the estimation of motor improvement in subacute stroke patients. Moreover, the temporal dynamics of the performance measures (Fig. 4a) appeared to be similar to the ones previously reported for chronic stroke patients [29, 41]. In past studies, the selected measures have been shown to correlate with clinical scores [48] and they have been linked to distinct post-stroke deficits and mechanisms of recovery [41, 49]. Specifically, the percentage of accomplished tasks was mostly associated to paresis (i.e., the decreased ability to volitionally modulate motor units activation [50]), whereas movement velocity and smoothness were related to an abnormal muscle tone [49]. Although continuous adaptation of the difficulty for reaching tasks has been explored before [13, 21, 25], the decisions to change task difficulty were mainly based on one or multiple task completion variables, measuring whether the patient was able to complete tasks or not. The present study extends the decision rules by additionally integrating two variables related to movement kinematics, namely movement velocity and smoothness, which also characterized the neuro-biomechanical status of the patients [51]. Nevertheless, some tuning of the parameters could be considered to further enhance the efficacy of the motor improvement model. For instance, we observed that the patient with a lower degree of initial impairments (P02) barely made use of the robotic assistance provided by the exoskeleton, leading to almost no variance in the variable SUCC. In this regard, future studies may explore other performance measures and models, to achieve a more exhaustive evaluation of the patients’ status. In this context, the use of a model-based approach, such as the one proposed in the current work, can facilitate the integration of other measures which have been explored before, such as for example muscle activity [18] or psychophysiological signals [28].

The results for the two patients showed as well that targets were replaced by the model at appropriate moments, i.e., when the patients’ performance had improved and started to saturate. Indeed, it could be argued that a replacement of a subtask occurring too soon would have led to degraded motor performances in further evaluations. However, motor performances of both patients were retained when targets were reintroduced (Fig. 4c), indicating that the estimated recovery was preserved. Nevertheless, other methods for task scheduling could be introduced to further optimize the training progression. Indeed, previous work has suggested that effective scheduling of multitask motor learning should be based on prediction of long-term gains rather than on current performance changes [52]. Along these lines, we have implemented the time window of the last four repetitions, which are always taken into account for the evaluation of motor performance. However, it should be acknowledged that other, more sophisticated, methods to adapt the schedules may lead to higher gains in rehabilitation and are therefore worth exploring. For instance, task difficulty could be increased by introducing new subtasks depending on more complex movements within the same workspace, in order to exploit generalization effects [53, 54]. Another possible approach could be a semi-automatic implementation of the training adaptation, where the physical therapists remains in charge of the task adaptation, in order to benefit from their expertise, while in parallel harnessing the real-time MI estimates provided by the model as a decision support. Such solutions could further improve engagement and enhance the rehabilitative treatment by providing training tasks specifically adapted to the ability level of the patient.

The retention of improvements at target reinsertion together with the increase in FMA-UE scores for both patients are promising indications for the usability and efficacy of the presented approach in clinical settings. Nevertheless, it has to be acknowledged, that it is also known that subacute patients often report motor improvements even with limited training [55]. Therefore, at the current state of this research, it cannot be presumed that improvements were merely elicited by the adaptive robot-aided therapy. However, several pieces of evidence suggested that the period immediately after the lesion, normally characterized by spontaneous neurological recovery, represents the critical time window in which the delivery of high dose and intense neurorehabilitation can elicit crucial improvements in functional tasks [56, 57]. Therefore, more and more robot-aided rehabilitation trainings should be targeting subacute stroke populations. In this context, our results illustrate the feasibility of using a personalization method to continuously monitor the status of both mild and severely impaired subacute stroke patients and to automatically adapt their motor retraining within practice sessions by continuously challenging their neuromuscular system.

### Limitations of the study

Although the results of this study suggest that the proposed approach might be interesting for clinical applications, the limited sample size as well as the lack of an experimental control group receiving standardized robotic therapy, constrains the generalizability of the reported results. Yet clinical efficacy was not probed in this work. Moreover, the very different severities of initial impairment observed in the two patients of the pilot study do not allow for a controlled comparison. Therefore, further studies including larger cohorts of participants would be necessary to draw meaningful conclusions about the clinical relevance of the presented approach. Yet the results obtained from the present study may provide a useful basis for the design and implementation of such clinical studies. In this context, it would be particularly interesting to compare the clinical outcomes of the personalized approach presented in this study with non-adaptive robotic or conventional rehabilitation trainings. This is important, since previous work has suggested that pseudo-random scheduling of multiple tasks may be almost as effective as adaptive scheduling approaches [52].

## Conclusions

In this work, we presented a model-based approach to personalize robot-aided rehabilitation therapy within rehabilitation sessions. The feasibility of this approach was demonstrated in experiments with seventeen healthy subjects and a pilot test with two subacute stroke patients providing promising results. However, due to the limited sample size, larger studies would be needed to demonstrate clinical relevance of the presented approach. While we implemented the proposed method for the use in upper limb rehabilitation of stroke patients, the usage is certainly not limited to such applications. The presented model can be adapted for the use with other robotic rehabilitation devices and training tasks, exploiting different performance measures and/or different observation equations. The real-time functionality and the identification of subject-specific abilities at subtask level could enhance robot-aided rehabilitation training, making it more purposive and efficient for the patients.

## Methods

In the current study, we developed a model to continuously estimate motor improvement (MI) in three-dimensional workspaces using kinematic performance measures, based on the results of our previous work [29]. Moreover, we designed a personalization routine, that automatically adapts the difficulty of the rehabilitative motor task (i.e., a point-to-point reaching task) based on the MI estimates. Both the MI model and the personalization routine were integrated in the control algorithm of an upper-limb exoskeleton and tested with a group of 17 healthy participants. The presented approach was then tested with two subacute stroke patients.

### Participants

#### Healthy participants

Seventeen right-handed subjects (eight males, nine females, 25.4 ± 3.3 years old) participated in the experimental validation of our approach. The participants did not present any evidence or known history of skeletal and neurological diseases and they exhibited normal ranges of motion and muscle strength. All participants gave their informed consent to participate in the study, which had been previously approved by the Commission Cantonale d’Éthique de la Recherche Genève (CCER, Geneva, Switzerland, 2017-00504).

#### Subacute stroke patients

Two subacute stroke patients from the inpatient unit of the Hôpitaux Universitaires de Genève (HUG, Geneva, Switzerland) were included in the study. A summary of the patient information is reported in Table 4. Both patients suffered from a right hemiplegia with at least 10° of residual motion in shoulder and elbow joints. The patients were enrolled in the study within 2–8 weeks after the stroke. Both patients received standard therapy at the stroke unit during the acute phase, and an individually tailored multidisciplinary rehabilitation program in the subacute and chronic phases. The patients received two times 30 min of physical therapy per day on 5 days per week and five times 30 min of occupational therapy per week on an inpatient basis for 8 to 16 weeks, followed by outpatient treatment of 1 to 4 h of physical and occupational therapy per week. Therapy was adapted by the therapists to the current capacities of the patients by choosing from a list of appropriate exercises comprising upper-extremity relaxation techniques, unilateral task-specific mobilizations, bilateral upper limb exercises with a wand, ball exercises, active ante/retropulsion exercises, active pronation/supination exercises and grasping exercises. Therapists were assigned to the patients based on their availability; hence different therapists took care of the patients throughout the therapy sessions. In addition to the standard therapy, the patients received robot-aided treatment following the adaptive robotic rehabilitation protocol described in the “Subacute stroke patients” section. All patients gave their informed consent to participate in the study. This study is registered in ClinicalTrials.gov (NCT02770300) and the experimental protocols were approved by Swissmedic and Swissethics.

**Table 4:** Demographics and information of the stroke patients included in the study

Patient	Gender	Age	Weight (kg)	Height (cm)	Hand dominancy	Stroke diagnosis	Enrolment after lesion
P01	Male	86	66	165	Right	Ischemic, middle cerebral artery left, cerebellum right	3 weeks
P02	Male	65	81	180	Right	Ischemic, corona radiata left	2 weeks

### Robotic exoskeleton and motor task

We implemented the motor improvement model and the personalization routine in the robotic upper-limb exoskeleton ALEx (Wearable Robotics srl. [58, 59]). During the experiments, the patients and the healthy participants were instructed to perform point-to-point reaching movements at their comfortable velocity (Fig. 1a). All reaching movements started from the center of the workspace and the goal was to reach one of the 18 targets equally distributed over the three planes of a sphere of 19 cm of radius (Fig. 1b). The selected radius of the sphere allows for a maximum exploration of the workspace, while maintaining the reaching movements executable for people of most body sizes. Each movement towards a target represented a subtask. This design allowed exploiting an extensive three-dimensional workspace and provided means to easily identify all subtasks of the exercise. The sphere was positioned so that its center was aligned with the acromion of the right arm mid-way between the center of the target panel and the subject’s acromion. The targets were displayed on a screen mounted in front of the subjects and visual feedback was provided by means of a cursor mapping the position of the exoskeleton’s handle to the screen. In order to preserve the depth perception, the dimensions of the target spheres were modified in accordance with their position in the 3D space. If a subject was unable to reach a target (i.e., the subject did not move for more than 3 s), ALEx activated its assistance mode to guide the subject towards the target according to a minimum jerk speed profile [60].

### Experimental protocols

#### Healthy participants

The healthy participants attended a single experimental session, which comprised seven blocks of reaching movements (Fig. 1c). Breaks of 1 min were scheduled after each block to prevent fatigue. The session started with an initial assessment block consisting of three runs (A_I,1–3_). During each run all 18 targets were presented once and in a randomized order. The purpose of the assessment block was (i) to allow familiarization with the robotic system and the motor task and (ii) to record a baseline for the performance measures. This block was followed by five blocks B_1–5_ during which the visual feedback was inverted (i.e., an upward movement was displayed as downward and vice versa, likewise for left/right and forward/backward movements). This vision inversion was introduced to induce motor performances with temporal dynamics resembling the ones observed in robot-aided rehabilitation of stroke patients [29, 36, 37]. At the onset of the five inversion blocks, participants were not informed about the inversion of the visual feedback, but they were told that the task difficulty was changed. Each of the five inversion blocks B_1–5_ consisted of five runs, each one composed of eight point-to-point reaching movements for a total of 40 reaching movements per block.

The initial set of training targets for each participant was generated following a semi-randomized procedure: based on the hypothesis presented in the Introduction, we expected movements towards on-axis targets (i.e., targets 1, 3, 5, 7, 10 and 13, see Fig. 1b) to be easier. Therefore, the initial set of training targets always contained all six on-axis and two randomly selected off-axis targets (i.e., targets 2, 4, 6, 8, 11, 14, 15, 16, 17 and 18). The presentation order of the eight initial training targets was randomized. The remaining ten off-axis targets were placed randomly in the training queue.

During the five inversion blocks B_1–5_, MI was continuously estimated for each training target and a target was removed from the current set of training targets if the MI estimates for this subtask satisfied the replacement conditions (see “Training adaptation routine” section). In this case, the target was replaced by the next one in the training queue. The inversion blocks B_1–5_ were followed by a final assessment block which was composed of three runs (A_F,1–3_) and followed the same procedure as the initial assessment block (i.e., neither vision inversion nor training adaptation were applied). The data acquired during the assessment blocks (i.e., A_I,1–3_ and A_F,1–3_) were not considered for the motor improvement estimation.

#### Subacute stroke patients

The experimental protocol for the patients consisted of 4 weeks of robot-aided rehabilitation therapy (Fig. 1d), with three sessions of 30 min per week. The training comprised the regular point-to-point reaching task (see “Robotic exoskeleton and motor task” section). In order to evaluate the outcome of their rehabilitation training, the patients completed two assessment sessions before (A_I,1–2_) and after (A_F,1–2_) the therapy. The initial assessment sessions A_I,1–2_ were completed 2 weeks and 1 week before the beginning of the therapy. The final assessment sessions A_F,1–2_ were completed 1 week and 1 month after the end of the therapy. During the initial and final assessment sessions, all eighteen targets of the point-to-point reaching task were presented to the patients in a randomized order. The total amount of reaching movements for each session was determined by the physical therapist while encouraging the patient to perform as many movements as possible (numbers reported in “Pilot test with subacute stroke patients” section). Breaks of varying durations were scheduled based on the patient’s condition. In addition, the patients were evaluated using the upper extremity section of the Fugl-Meyer assessment (FMA-UE, [61]). The data acquired during the assessment blocks (i.e., A_I,1–2_ and A_F,1–2_) were not considered for the motor improvement estimation.

For the treatment sessions, we first identified the patient-specific difficulty for each of the 18 targets following the initial assessment sessions A_I,1–2_. Specifically, we analyzed the mean values of the performance measures MV, SAL and %SUCC (see “Performance measures” section) for each of the 18 training targets. The targets were first ordered by descending values of %SUCC (i.e., starting from easier targets). If several targets had equal values for %SUCC, the order amongst them was determined by their mean values for MV and SAL, while giving both measures equal weight. The first eight targets of the resulting list were selected as the initial training targets. The remaining targets were placed in a training queue while conserving the determined order of difficulty. During the therapy (*W*_1_–*W*_4_, Fig. 1d), MI was continuously estimated for each training target separately. The replacement of a training target based on the MI estimates followed the procedure presented in “Training adaptation routine” section. The current set of training targets was saved after the completion of each training session, ensuring continuity between sessions. The total amount of reaching movements for each session was determined by the physical therapist while encouraging the patient to perform as many movements as possible. However, no decisions were taken by the therapist regarding the choice of the specific training targets. Breaks of varying durations were scheduled based on the patient’s condition.

### Motor improvement model

In order to continuously track patients’ MI at subtask level (i.e., for a series of point-to-point reaching movements in different directions), we used a state-space model. MI was modeled as a random walk:

1 $${\text{MI}}_{k} = {\text{MI}}_{k - 1} + \epsilon_{k} ,$$

where *k* are the different repetitions for a movement direction and $\epsilon_{k}$ are independent Gaussian random variables with zero mean and variance $\sigma_{\epsilon }^{2}$. A set of observation equations *z*_*j,k*_ was defined in order to estimate MI. These equations related MI to continuous performance measures *r*_*j*_, which were computed from kinematic recordings provided by the robotic device (see “Performance measures” section for details on the performance measures). The continuous variables *r*_*j*_ (with $j = 1, \ldots ,J$ representing the different performance measures) were defined by the log-linear probability model

2 $$z_{j,k} = \log \left( {r_{j,k} } \right) = \alpha_{j} + \beta_{j} {\text{MI}}_{k} + \delta_{j,k} ,$$

where $\delta_{j,k}$ are independent Gaussian random variables with zero mean and variance $\sigma_{\delta ,j}^{2}$. The use of log-linear models allowed capturing rapid increases (or decreases) of the performance measures during the training, as well as the expected convergence towards subject-specific upper (or lower) bounds at the end of the training. The suitability of such probability models for motor performance measures in stroke patients was previously demonstrated [29, 41]. Similarly, an observation equation for a discrete performance measure *n*_*k*_ was defined. The binary discrete variable *n*_*k*_∈ {0, 1} was used to track the completion of the exercised subtask, with 1 meaning that the subtask was performed successfully and 0 meaning failure. The observation model for *n*_*k*_ was assumed to be a Bernoulli probability model:

3 $$\Pr (n_{k} |p_{k} ) = p_{k}^{{n_{k} }} \left( {1 - p_{k} } \right)^{{1 - n_{k} }} ,$$

where *p*_*k*_, the probability of performing the subtask successfully at repetition *k*, was related to MI_*k*_ by a logistic function:

4 $$p_{k} = \frac{{\exp \left( {{\text{MI}}_{k} } \right)}}{{1 + \exp \left( {{\text{MI}}_{k} } \right)}}$$

ensuring that *p*_*k*_ was constrained in [0, 1]. Furthermore, this formulation guaranteed that *p*_*k*_ would approach 1 with increasing MI.

The model parameters {*α*_*j*_, *β*_*j*_, *σ*_*δ,j*_, *σ*_*ϵ*_, *p*_*k*_} were estimated for each individual subject using the recordings of *r*_*j,k*_ and *n*_*k*_ (i.e., kinematic recordings from the robotic device, see “Performance measures” section) and by applying Bayesian Monte Carlo Markov Chain methods. The estimation of the parameters resulted in an estimate for MI. In order to ensure accuracy of the model, it was necessary that the number of recordings of *r*_*j,k*_ and *n*_*k*_ exceeded the number of parameters. Based on simulations performed with varying number of data points (detailed description can be found in Additional file 1), the minimum number of data points for MI estimation was set to 8. In order to validate the capability of the proposed approach to appropriately capture variable dynamics of the performance measures, we simulated different rehabilitation scenarios under varying conditions (see Additional file 1). As we aimed at estimating MI at subtask level, separate MI models were used for each movement direction of the training exercise.

### Performance measures

Previous studies have shown that mechanisms of post-stroke recovery can be described by factors related to movement velocity, smoothness, and efficiency [29, 41, 48]. Unlike physiological signals, these kinematic performance measures can be easily recorded and processed in real-time, promoting their use in clinical settings. In this study, we selected two continuous performance variables *r*_*j*_ for the use with the MI model: (i) the mean velocity of a movement (MV) and (ii) the spectral arc length (SAL), a robust and consistent measure of movement smoothness [62]. MV was calculated using the *x*, *y*, and *z* coordinates of the robotic handle recorded by the exoskeleton for each movement. SAL is a dimensionless measure quantifying movement smoothness by negative values, where higher absolute values are related to jerkier movements. It was calculated using the position of the robotic handle recorded by the exoskeleton for each movement and the mathematical equations presented in [62]. Regarding rehabilitation training, values of SAL close to zero are desirable, as well as high values of MV. The discrete variable *n*_*k*_, instead, was denoted as success (SUCC) and defined separately for the experiments with the healthy participants and the patients. For the patients, the value of SUCC was determined by the robotic assistance (i.e., SUCC = 1 if the patient performed the movement without robotic assistance, SUCC = 0 otherwise). On the other hand, the healthy participants were expected not to rely on the robotic assistance, although it was also provided if necessary. This assumption was supported by preliminary experiments with healthy subjects (see Additional file 1). Therefore, in order to have an equivalent discrete variable for the experiment with healthy subjects, we defined the value of SUCC based on the execution time (i.e., SUCC = 1 if a healthy participant completed the movement within the time threshold *t*_th_, SUCC = 0 otherwise). The time threshold *t*_th_ was set to 4 s based on preliminary experiments with healthy subjects (see Additional file 1).

### Training adaptation routine

Using the model described in the previous section, MI was continuously tracked for each subtask (i.e., a movement towards a specific target) and used to implement a personalized training routine (Fig. 1e). At the beginning of the training, we identified the subject-specific difficulty level for each subtask of the training exercise based on an initial assessment of the performance measures. The subtasks were then ordered by increasing difficulty and the easiest ones were selected as the initial training set. During the training, a subtask was removed from the set of current training subtasks when the MI estimates for this movement exceeded a given threshold and approached a plateau. Specifically, the probability of performing the subtask successfully *p*_*k*_, had to be greater than 0.5, and the difference between two consecutive MI values (i.e., between two repetitions of the same subtask) had to be smaller than 5% for at least four repetitions. Given the observation equation for *p*_*k*_, the former condition (*p*_*k*_ > 0.5) can be equally expressed in terms of the motor improvement: MI_*k*_ > 0. Once these conditions were satisfied, the subtask was replaced by a more difficult one from the training queue. The removed subtask was placed back into the training queue, so that it could be reintroduced at a later stage.

### Statistical analysis

Healthy participants were grouped into fast (*n* = 9) and slow (*n* = 8) adapters by a post hoc median split based on the total number of replaced targets during the inversion blocks.

For the healthy subjects, statistical tests were performed to support the following hypotheses:

i. The introduction of the vision inversion degrades the performance of fast and slow adapters for the reaching movements.
ii. Performances for reaching movements with vision inversion improve with training for both groups.
iii. Even after training, performances for reaching movements with vision inversion of both groups do not reach the levels of the initial assessment (without inversion).
iv. There is a performance difference between fast and slow adapters, which is observable in the moment the vision inversion is introduced and also at the end of the vision inversion.

For the blocks A_I,1–3_, B_1_ and B_5_, movements towards all presented training targets were combined to calculate mean values for MV, SAL and %SUCC for each healthy subject. Each individual performed 54 movements in A_I,1–3_ and 40 movements in B_1_ and B_5_. Using the Shapiro–Wilk normality test, mean values for MV, SAL, %SUCC for fast and slow adapters were tested for normal distribution separately. The data were not normally distributed. In order to support the first three claims, a Friedman test was performed for the mean values of MV, SAL and %SUCC for the three time blocks (i.e., for A_I,1–3_, B_1_ and B_5_). Following the Friedman tests, post hoc tests were performed between pairs of blocks using Wilcoxon signed-rank tests with Holm–Bonferroni correction (for three comparisons) to illustrate the differences between the blocks. Specifically, point (i) was verified by comparison between A_I,1–3_ and B_1_; point (ii) was verified by comparison between B_1_ and B_5_; and point (iii) was verified by comparison between A_I,1–3_ and B_5_. To verify point (iv), we used Wilcoxon rank-sum tests with Holm–Bonferroni correction (for three comparisons) to compare the mean values of MV, SAL and %SUCC between fast and slow adapters in the blocks A_I,1–3_, B_1_ and B_5_.

For the patients, statistical tests were performed to support the following hypotheses:

i. Motor performance in the reaching task improved for both patients following the completion of the robot-aided rehabilitation training.
ii. The motor improvement is retained 4 weeks after the end of the training for both patients.
iii. Motor performance right before the replacement of a target and after reinsertion of the same target is comparable.

For the assessment blocks, all movements performed by the patients in the session were combined, resulting in 36 observations for P01 in each block and 54 observations for P02, respectively. To verify the two first claims, Friedman tests were performed for MV, SAL and %SUCC between the initial assessment session right before the treatment sessions (A_I,2_), the final assessment session right after the training (A_F,1_), and the follow-up session (A_F,2_). Following the Friedman test, pairwise comparisons were performed, between these blocks using Wilcoxon signed-rank tests with Holm–Bonferroni correction (two comparisons). Specifically, we compared A_I,2_ and A_F,1_ to verify claim (i) and A_F,1_ and A_F,2_ to verify claim (ii). Friedman tests and pairwise comparisons were performed for each patient separately. To verify claim (iii), we analyzed the values of MV, SAL and %SUCC before the replacement of the targets and after their reinsertion. For each target, mean values of improvement were calculated with respect to the first four repetitions in A_I,2_ for the last four repetitions before replacement and for the first four repetitions after reinsertion. We then used Wilcoxon signed-rank tests to analyze differences in MV, SAL and %SUCC for all targets before replacement and after reinsertion. The analyses were performed for each patient separately. The power of the statistical tests was computed using *z*-tests and approximations of normal distributions of the data. All analyses were performed using MATLAB (The MathWorks, Natick, Massachusetts). The significance levels were set to alpha < 0.05 (type I error) and beta < 0.2 (type II error).

## Supplementary information

**Additional file 1.** Supplementary analysis of performance measures and model parameters.

## Data Availability

Since the data used in this study include data collected in a clinical trial with patients, the data will not be shared.

## Abbreviations

MI: Motor improvement
MV: Movement velocity
SAL: Spectral arc length
SUCC: Success variable for a single movement
%SUCC: Success rate for a series of movements
FMA-UE: Fugl-Meyer assessment for upper extremities
